# Comparative Therapeutic Effects of Velaglucerase Alfa and Imiglucerase in a Gaucher Disease Mouse Model

**DOI:** 10.1371/journal.pone.0010750

**Published:** 2010-05-20

**Authors:** You-Hai Xu, Ying Sun, Sonya Barnes, Gregory A. Grabowski

**Affiliations:** 1 Division of Human Genetics, Cincinnati Children's Hospital Research Foundation, Cincinnati, Ohio, United States of America; 2 Department of Pediatrics, University of Cincinnati, Cincinnati, Ohio, United States of America; National Institutes of Health, United States of America

## Abstract

Gaucher disease type 1 is caused by the defective activity of the lysosomal enzyme, acid β-glucosidase (GCase). Regular infusions of purified recombinant GCase are the standard of care for reversing hematologic, hepatic, splenic, and bony manifestations. Here, similar *in vitro* enzymatic properties, and *in vivo* pharmacokinetics and pharmacodynamics (PK/PD) and therapeutic efficacy of GCase were found with two human GCases, recombinant GCase (CHO cell, imiglucerase, Imig) and gene-activated GCase (human fibrosarcoma cells, velaglucerase alfa, Vela), in a Gaucher mouse, D409V/null. About 80+% of either enzyme localized to the liver interstitial cells and <5% was recovered in spleens and lungs after bolus i.v. injections. Glucosylceramide (GC) levels and storage cell numbers were reduced in a dose (5, 15 or 60 U/kg/wk) dependent manner in livers (60–95%) and in spleens (∼10–30%). Compared to Vela, Imig (60 U/kg/wk) had lesser effects at reducing hepatic GC (*p* = 0.0199) by 4 wks; this difference disappeared by 8 wks when nearly WT levels were achieved by Imig. Anti-GCase IgG was detected in GCase treated mice at 60 U/kg/wk, and IgE mediated acute hypersensitivity and death occurred after several injections of 60 U/kg/wk (21% with Vela and 34% with Imig). The responses of GC levels and storage cell numbers in Vela- and Imig-treated Gaucher mice at various doses provide a backdrop for clinical applications and decisions.

## Introduction

In Gaucher disease, defective acid β-glucosidase (GCase) activity leads to accumulation of its substrate, glucocerebroside (glucosylceramide [GC]), in lysosomes of macrophages in many organs. This results in the tissue appearance of characteristic storage cells, Gaucher cells [1], [2]. The pathologic accumulation of such storage macrophages leads to the major manifestations including hepatosplenomegaly, blood cytopenias, and bone marrow abnormalities [1]. Deduve [3] was the first to suggest that reconstitution of lysosomal enzyme activities could lead to clinical improvement. Biochemical evidence to support this notion was derived from *ex vivo* cross-correction of cells from patients with genetically distinct mucopolysaccharide storage diseases in which intracellular storage was reduced by cross-correcting soluble factors (enzymes) [4]. Based on the concepts of receptor-mediated endocytosis through carbohydrate recognition receptors, enzyme replacement/reconstitution therapy became a reality for Gaucher disease [5], [6], [7], [8], using mannosyl-terminated human placental GCase (alglucerase). Small clinical trials showed improvement in the clinical and biochemical features of the disease [5]. Later, recombinant α-mannosyl-terminated human GCase (imiglucerase, Imig) was developed and was shown to have biologic and therapeutic equivalency to alglucerase [6], [9]. This therapy has become the standard of care for significantly involved patients with Gaucher disease type 1 [8].

Enzyme replacement therapy (ERT) has dramatically altered the visceral phenotype of Gaucher disease and improved the overall disease course in afflicted people [6], [7], [8]. For many affected people the regular use of ERT improves the hepatosplenomegaly within two years, accompanied by improvements in anemia and thrombocytopenia [10]. Improvements in bone density [11], [12], bone pain, and crisis of avascular necrosis also occur [13]. ERT also can restore normal growth patterns in the ∼35% of children with Gaucher disease and growth retardation [14]. Since 1991, >5,000 individuals with Gaucher disease type 1 have received regular infusions of α-mannosyl-terminated human GCase [5], [6], [10], [15], [16], [17]. A variety of doses and dosage schemes had varying degrees of efficacy in hepatic, splenic, and bone marrow involvement [10], [16], [18]. Detailed analyses of patients statistically matched for phenotype demonstrated an incremental therapeutic dose response with Imig, thereby providing data to facilitate personalization of dosing regimens [18], [19]. These advances have been based primarily on clinical outcome measures of visceral and hematologic resolution, with little data about the pharmacology [20], [21], tissue distribution, or cellular localization in the target organs [22], [23]. Histological and enzyme data in patients are scarce due to the invasive nature of tissue sampling and the inaccessibility of most tissues for systematic analyses. From a few *in vivo* and autopsy studies, significant amounts of enzyme were apparent in hepatic and/or splenic tissues for several days after enzyme injection, with very small amounts detected in the lungs and bone marrow mononuclear cells [15], [24]. These results, coupled with organ-specific therapeutic guidelines [25] provide additional guidance for patients and their physicians and for new innovative, adjunctive, and competitive therapies.

To date, most ERT data for Gaucher patients were obtained from the use of Imig treatment. Imig is human recombinant GCase that is secreted from Chinese hamster ovary (CHO) cells with attached complex N-linked oligosaccharides. The purified enzyme is then sequentially deglycosylated to expose ∼3 α-mannosyl residues on short N-linked oligosaccharide chains [26]. This modified enzyme has preferential distribution to and uptake into macrophages via the macrophage mannose receptor [21]. In addition, Imig has a single amino acid difference from the natural sequence, by containing a histidine at residue 495 rather than an arginine. Recently, GCase has been produced by gene activation in a human fibrosarcoma cell line (velaglucerase alfa, Vela). To achieve α-mannosyl residue exposure, these cells are treated with kifunensine, an inhibitor of the α-mannosidase I that is present in the endoplasmic reticulum [27]. This treatment leads to a GCase with higher α-mannosyl content than the CHO-derived GCase, since the natural sequential remodeling of the N-linked oligosaccharides during transit through the Golgi is inhibited/prevented [27]. In addition, Vela has the wild type sequence with an arginine at position 495. Previously, the exchange of the histidine and arginine at position 495 was shown to have no effect on any *in vitro* physicokinetic properties [9], [28], or on the crystal structure [17], [29].

In general, ERT with GCase has a low infusion-related adverse event profile [30], [31]. Many of these are antibody (usually IgG) mediated, and are managed by decreasing drug delivery rates or cotreatment with antihistamines or, occasionally, corticosteroids. In Gaucher disease, about 13–15% of patients developed an IgG antibody response to alglucerase or Imig [32], whereas antibody conversions were from 50–91% for the respective ERTs in other lysosomal diseases [30]. Documented IgE-mediated or anaphylactic reactions with Imig have been very infrequent (<1%), and some patients were able to resume ERT following desensitization or by using ramp-up infusion protocols [31].

The availability of mouse models that are analogues of visceral Gaucher disease in humans allows for direct *in vivo* comparison of the PK/PD and therapeutic effects [33] of different ERTs or other therapeutic agents. Here, the biochemical properties, PK/PD, and therapeutic effects of Vela and Imig were directly compared at multiple doses. These studies also showed a complex uptake and disappearance of exogenously administered enzyme that resulted in a non-uniform tissue and cellular delivery of active enzyme.

## Results

### *In vitro* enzymology

#### GCase activity

Catalytic activities of Vela and Imig were analyzed with 4MU-Glc and GC substrates. Vela had ∼6–10% reproducibly greater activity compared to Imig (Table 1). The functional active sites of these two enzymes were quantified using the single turnover substrate DNPFG, and consistent with the hydrolytic activity, ∼5–7% more functional active sites were quantified with Vela than with Imig (Table 1). The respective K_m_ values for the 4 MU-Glc were 1.59±0.2 or 1.65±0.25 mM (n = 6, *p* = 0.850).

**Table 1:** Vela and Imig active sites and specific activities.

Source	Active sites	GCase activity (nmole/h/mg)	
	2,4-DNPFG (n = 8)^A^	NBD-GC (n = 10)^A^	4MU-Glc (n = 6)^A^
Imig	91.6±2.2%	1,191,610±56,390	966,000±8,467
Vela	97.4±0.6%	1,321,057±67,237	1,030,480±2,692

A mean ± SE, Active sites are expressed as mole ratios of 2,4-DNP released and enzyme protein.

#### Interaction of GCase and active site-directed inhibitors

The reversible competitive and covalent active site-directed (Conduritol B epoxide, CBE) inhibitors of GCase showed comparable interaction with both enzymes [34], [35], [36] (Table 2). IFG binding is of interest, since crystallization studies show that this agent induces a significant conformational change and enhanced enzyme stability [35]; similar changes are implied by comparable K_i_ values. The IC_50_ or K_i_ values were similar for inhibitors of Vela and Imig (*p*>0.05). The comparative pH profiles of these two enzymes were similar in the range from pH 4.4–7.2. The peak activities were at pH = 5.4–5.6 (data not shown).

**Table 2:** Ki and IC_50_ values of GCase inhibitors for Vela and Imig.

Inhibitor	Vela	Imig	*p* value
Deoxynojirimycin (µM)*	23.4	27.4	0.8343
C4-Deoxynojirimycin (µM)	92.8	93.3	0.9692
C9-Deoxynojirimycin (nM)	88.1	66.9	0.8838
C12-Deoxynojirimycin (nM)	9.97	14.7	0.6957
Castanospermine (µM)	2.57	2.28	0.9972
Isofagomine (nM)	30.7	28.8	0.904
Conduritol B epoxide(µM)[IC_50_]	99.76	146.2	0.8775

*K_i_ values for all, except Conduritol B epoxide. Equal amounts of Vela or Imig protein (8 pmol) were used. SE in all measurements was ≤20%.

#### pH- and thermostabilities

The time-dependent stabilities of Vela and Imig were evaluated at various temperatures and pH values, as well as in either human or mouse serum. At pH = 7.4 in either citrate/phosphate buffer or mouse or human serum (buffered to pH 7.4) at either 37 or 25°C, both enzymes showed >50% irreversible losses of activity by 2 h; 70–85% losses were observed by 4 h under the same conditions (*p* = 0.99, Table 3).

**Table 3:** Thermostability of Enzyme Activity.

Condition	Vela, *t_1/2_* (min)	Imig, *t_1/2_* (min)	*p* value
25°C in CP, pH 7.4	118.6±1.91	122.4±7.75	0.6617
37°C in CP, pH 7.4	109.2±0.15	111.5±2.48	0.4223
25°C + h-serum	176.0±5.87	186.4±6.35	0.2562
25°C + m-serum	194.1±6.11	215.2±15.18	0.2273
37°C + h-serum	127.0±10.14	127.5±10.14	0.9747
37°C + m-serum	138.6±3.82	147.3±7.44	0.321

Vela or Imig activities were assayed as described in Methods. The *t*_½_ was the time at which GCase activity decreased to 50% of initial values. n = 3 to 6 for all assays.

#### Interaction with saposin C or BPS

Activation of Vela and Imig by the negatively charged lipid, BPS, and saposin C was determined in a detergent-free system with varying concentrations of either agent. The activities of both enzymes increased by 5- to 6-fold or 15- to 17-fold with saposin C (0 to 500 nM) or BPS (0 to 2 µM), respectively. In the presence of 0.5 µM of BPS either GCase showed saturation kinetics with a maximum activation by 200 nM saposin C. Similarly, in the absence of saposin C, both enzymes reacted maximal activation with 0.5 µM of BPS. No significant differences were found in these responses (*p* = 0.5875–0.8473).

#### Protease susceptibility

The susceptibilities of Vela or Imig to protease digestion were evaluated *in vitro* using Vela and Imig as substrates for cathepsin D [29]. Using equal amounts of Vela or Imig, cathepsin D (1 µg/ml) digestion led to ∼72–76% degradation of either enzyme after 4 h (data not shown). Both enzymes were completely degraded within 4 h using 2 µg/ml cathepsin D. There were no meaningful differences between the susceptibilities of either enzyme to cathepsin D digestion.

### Pharmacokinetics/pharmacodynamics

To determine the *in vivo* fate and stability of injected Vela or Imig, the disappearance of either enzyme was evaluated in serum and tissues. 9 V/null mice at 5 or 20 wks received intravenous boluses of 60 U/kg of Vela or Imig. Serum and tissues were harvested at 2 min to 42 h post-injection. Mice were perfused with saline prior to collection to clear tissues of blood that potentially contained exogenous GCase.

#### Serum GCase activities

**Figure 1:**
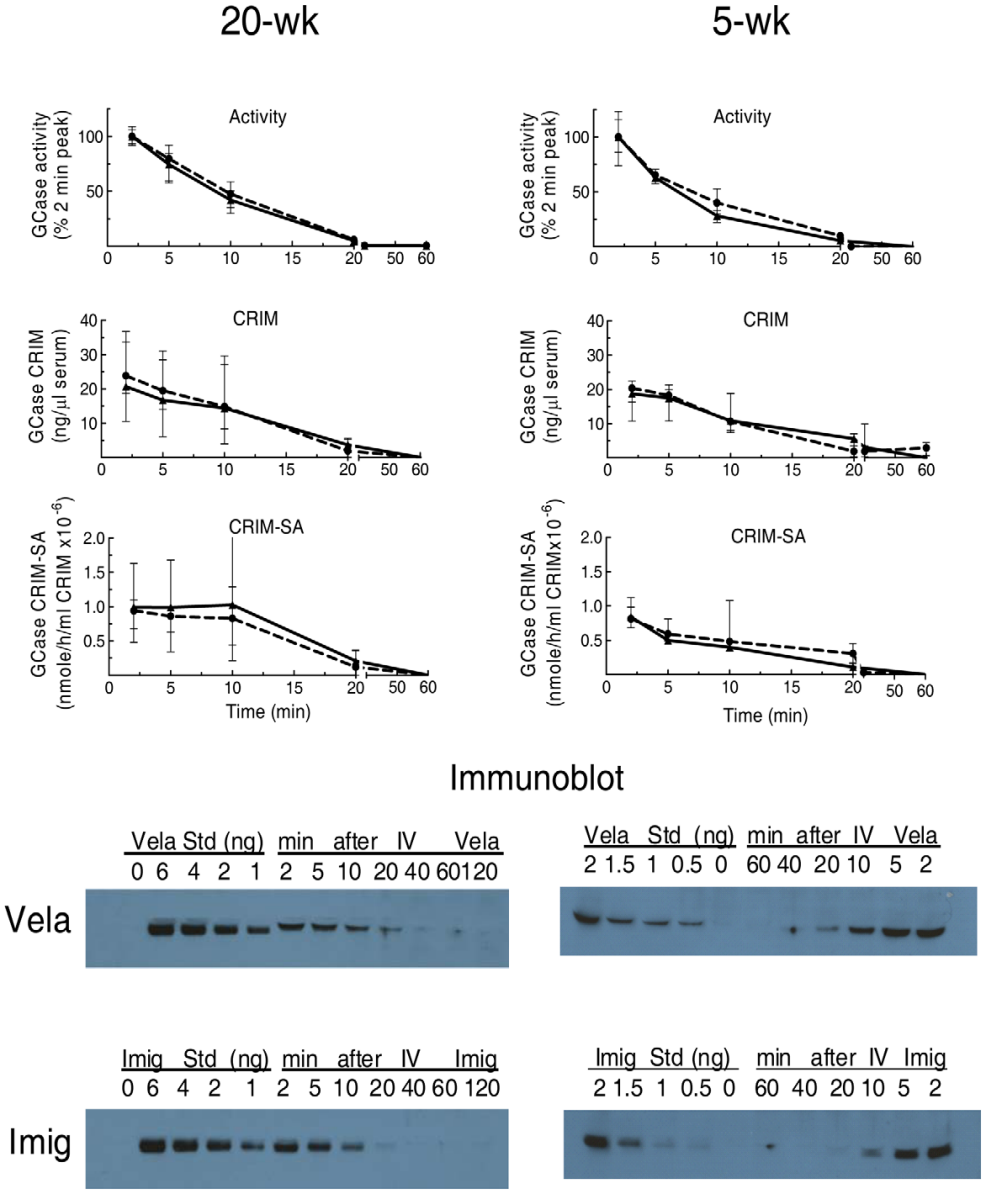
Serum disappearance of Vela or Imig in 20-wk (left) or 5-wk (right) 9 V/null mice. GCase activities in serum were measured at 2–60 min post-injection of Vela (▴, solid line) or Imig (•, dashed line). The values are referenced to the 2-min peak values. Serum disappearances of injected Vela and Imig proteins (CRIM panels) up to 60 min were analyzed by immunoblots (immunoblot panels). Known amounts of Vela or Imig protein (0.5–6 ng) were loaded on each blot as a reference for quantification of serum Vela or Imig protein for the respective GCases. CRIM-SA was the specific activity based on the amount of CRIM determined by corresponding immunoblots (CRIM-SA panels). Each data point represents the mean ± SE from duplicate assays of three mice in each cohort.

The GCase activities in serum from Vela- or Imig-injected 9 V/null mice (n = 3) were determined at each time point. Total blood volume in a mouse was estimated as ∼7% of body weight [37], and the serum yield is about 50% of blood volume. Based on these estimates, both 5- and 20-wk 9 V/null mice showed that about 60–65% of the injected enzyme (Vela and Imig) activities was recovered in serum at 2 min post-injection (Table 4). The disappearance of GCase activities in serum is presented as the percentage of 2 min values, as this was the earliest harvest time point possible. The clearance of both enzymes in serum had *t*_½_ = 8–11 min (Fig. 1). By 20 min post-injection, serum activities of injected Vela and Imig were <10% of the 2-min values. Concurrent GCase CRIM determinations in serum showed a similar disappearance profile, with *t*_½_∼13 min in both age groups (Fig. 1). When expressed as CRIM-SA, the decreasing values indicate losses of intrinsic catalytic activity during prolonged exposure to serum, so that by 20 min the intact enzyme remaining in serum has <10% of its original catalytic value. The enzymes' CRIM-SA appeared to be somewhat more stable in serum of 20-wk mice, but the SEs were large and this is not conclusive. In serum, GCase activities, CRIM, or CRIM-SA disappearances were similar for Vela and Imig at either 5 or 20 wks of age (*p*>0.05).

**Table 4:** Recovery of Vela or Imig activity in serum and organs of 9 V/null mice^A^.

Enzyme	Serum^B^		Liver^C^		Spleen^D^		Lung^C^	
	5-wk	20-wk	5-wk	20-wk	5-wk	20-wk	5-wk	20-wk
Imig	65.53±3.31	60.21±1.76	54.36±1.84	71.86±2.52	0.25±0.25	3.40±0.37	0.12±0.01	0.20±0.06
Vela	63.06±4.62	58.16±1.61	57.53±1.35	60.36±2.01	1.86±0.06	2.62±0.08	0.1±0.01	0.04±0.01

A The recovery of injected Vela or Imig was calculated as the percentage (% ± SE) of total serum or whole organ activity at the peak of enzyme activity following a bolus of 60 U/kg injection.

B The peak activity in serum was 2 min post injection.

C The peak activity in liver and lung was 20 min post injection.

D The peak activity in spleen was at 40 min in 5-wk mice and 20 min in 20-wk mice post injection.

#### Tissue GCase activities

**Figure 2:**
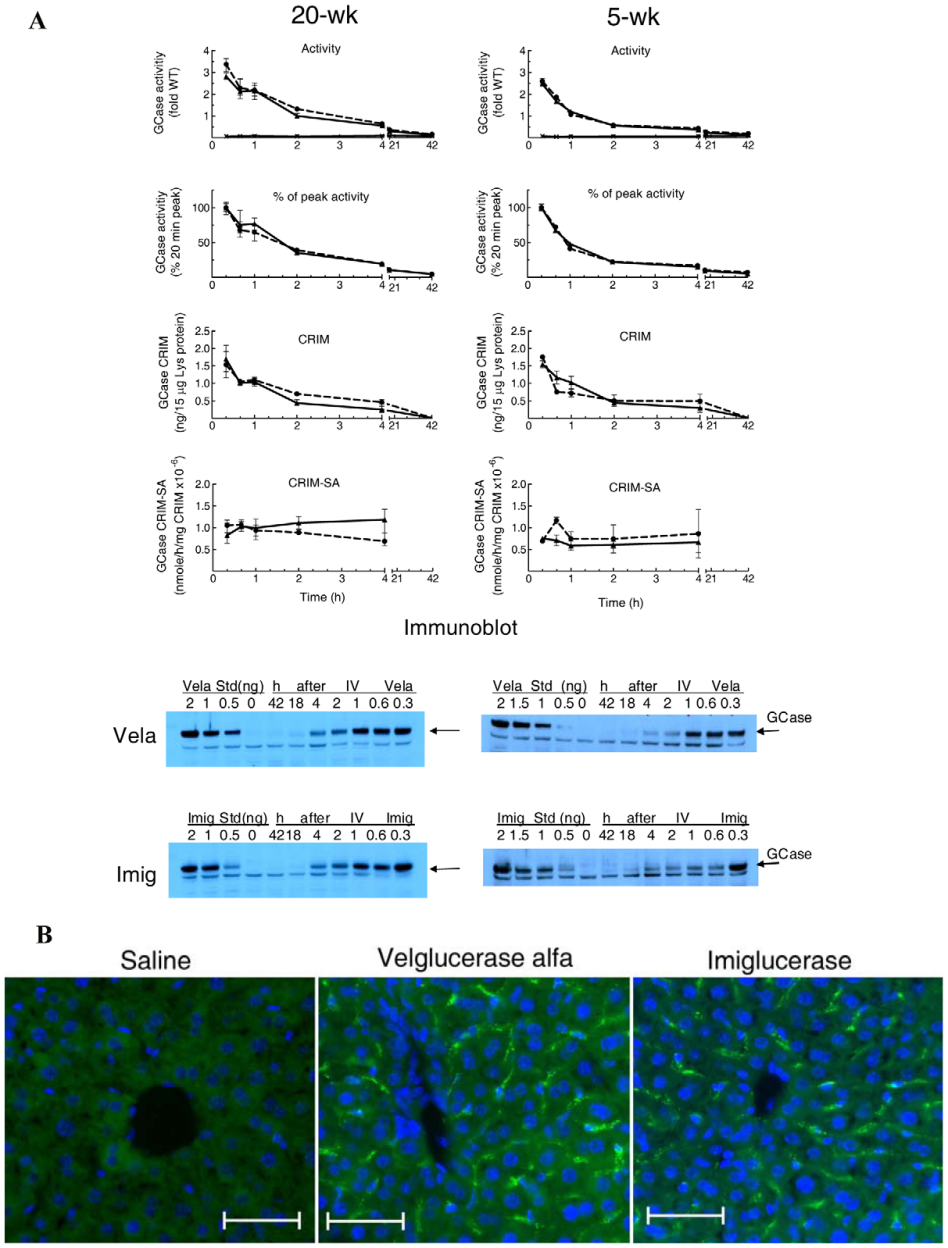
Fate and distribution of Vela and Imig in liver of 20-wk or 5-wk 9 V/null mice. (**A**) GCase activities in liver were assessed at 20 min to 42 h post-injection of Vela (▴, solid lines) or Imig (•, dashed lines). The results are referenced to average WT activity (fold change, WT = 1). The disappearance of Vela and Imig activities in liver were also plotted as the percentage of the 20-min peak values (% peak activity panels). The GCase activities in liver from saline-injected mice were 7.2% (SE = ±0.006) of WT (**X**, solid line at the bottom). The disappearances of the Vela and Imig protein (CRIM panels) up to 42 h post-injection were analyzed by immunoblots (bottom panels, arrows). To control for potential differential recognition by the rabbit anti-human GCase antibody, known amounts of Vela or Imig protein (0.5–2 ng) were loaded on each blot for quantification of Vela or Imig protein in liver against their respective standards. CRIM-SA is the specific activity per unit (ng) of CRIM determined by corresponding immunoblots. Each data point represents the mean ± SE from duplicate assays of three mice in each cohort. (**B**) Immunofluorescence of injected Vela or Imig in liver. Frozen liver sections from 9 V/null mice at 20 min post-injection of saline (left), Vela (middle), or Imig (right) were processed by immunofluorescence staining with rabbit anti-hGCase antibodies/goat anti-rabbit IgG-FITC. The hGCase signals (green) were located mainly in the periportal region of the liver acinus in the interstitial spaces between hepatocyte arrays. The magnification was 200×, scale bar  = 50 µm.

The uptake and disappearance of administered Vela or Imig were evaluated in liver, spleen, and lung. The total recovery of injected activity was based upon organ and body weights. In 20-wk 9 V/null mice, the peak liver activities (20 min) of injected Vela and Imig were 2.5- to 3.5-fold greater than WT levels, and they remained at or above WT levels for 3 h (Fig. 2A). GCase activity disappeared with t_½_∼100 min and decreased ∼30% within 40 min for either enzyme. This was followed by a slow decrease to 13–15% of WT by 42 h: this level of GCase activity was about twice that of saline-injected cohorts (7.2% of WT). The activity disappearance curves for Vela and Imig were very similar (*p* = 0.3048). The CRIM levels showed concordant disappearance profiles, with *t*_½_∼90–110 min for either GCase (*p* = 0.857) (Fig. 2A). In comparison to serum, the CRIM-SA's of the GCases were stable and did not differ between Vela and Imig (*p* = 0.46) for 4 h post-injection (Fig. 2A). At 18- or 42-h, the immunoblot signals were weak and not reliable. On the imunoblots, a non-GCase/non-specific, low molecular weight band was detected only in liver, including those with 0 ng of enzyme or at 42 h.

In 5-wk 9 V/null mice, these profiles were similar to those at 20 wks, with some differences. The peak activities (20 min) of Vela and Imig in liver in 5-wk mice were slightly less than those at 20 wks (∼2.5-fold of WT), and they remained at ≥WT level for ∼70–80 min post-injection, vs. ∼180 min in 20-wk mice. The activity disappearances of both enzymes in liver had a *t*_½_∼60 min (about 60% of the 20-wk mice), with a ∼60% drop within 40 min (∼2 times that of 20-wk mice). A slow decrease to 13–18% of WT occurred by 42 h (Fig. 2A). No differences in the activity disappearance curves were evident between Vela and Imig in the 5-wk livers (*p* = 0.9846). The CRIM disappearance curves paralleled those for GCase activity. The CRIM-SA was unchanged during this time course. The enzyme uptake into liver cells was evaluated by immunofluorescence at 20 min post-injection. Human GCase signals in liver from Vela- and Imig-injected mice were located mainly in the periportal region of the liver and in the interstitial spaces (Fig. 2B). These data indicate predominate uptake into Kupffer and sinusoidal endothelial cells of either GCase.

**Figure 3:**
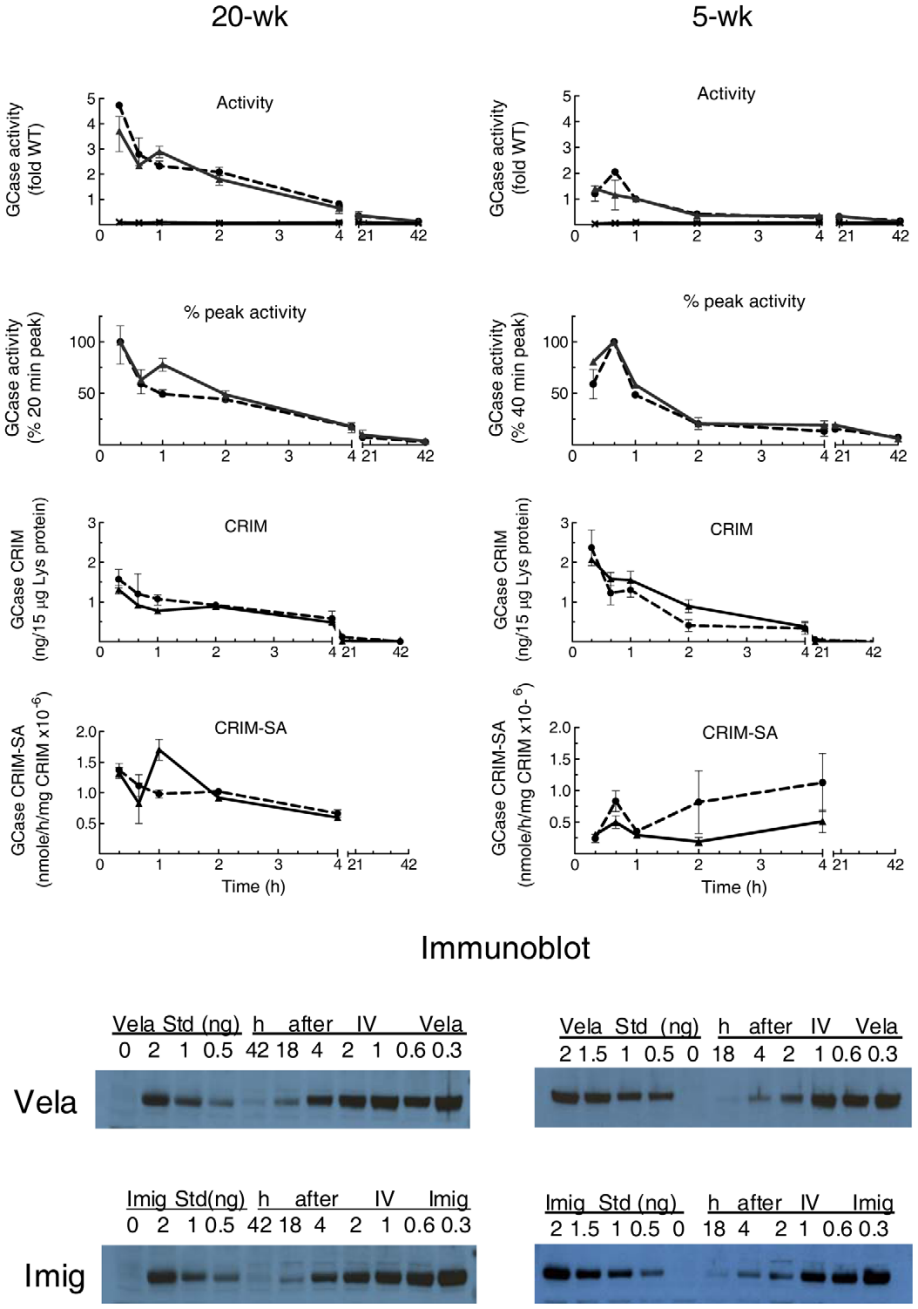
Disappearance of Vela or Imig in spleen of 20-wk (left) or 5-wk (right) 9 V/null mice. GCase activities in spleen were determined from 20 min to 42 h post-injection and represented as fold of WT (WT = 1) as in Figure 2. The disappearances of Vela or Imig activities were plotted as fold WT (activity panels) or from the 20-min (20-wk) or 40-min (5-wk) peak values (% peak activity panels). The GCase activities in spleen from saline-injected mice were 8.1% (SE = ±0.017) of WT (**X**, solid line at the bottom of top panels). Disappearances of Vela or Imig proteins (CRIM panels) up to 42 h post-injection were analyzed by immunoblots as per Figure 2.

The peak activities of Vela and Imig in spleen of 20-wk 9 V/null mice were 3- to 4.5-fold WT levels at 20 min post-injection, and they remained at ≥WT levels for ∼3.5 h. The enzyme activities decreased by ∼40% by 40 min and then smoothly returned to baseline activity over 42 h (Fig. 3). The GCase protein disappearance was concordant with the activity (*t*_½_∼1.0–1.5 h) for either enzyme (Fig. 3). The CRIM-SA of Vela or Imig in spleen was stable up to 4 h; by 18–42 h CRIM was not reliably detected. There were no significant differences between Vela and Imig for any of these parameters (*p* = 0.55–0.96).

In 5-wk 9 V/null mice, the peak activity in spleen was lower (by ∼2- to 3-fold) and later than in the 20-wk mice (Fig. 3). The activity disappearances of Vela and Imig were very similar (*p* = 0.761). The CRIM levels in spleen had a *t*_½_∼60 min (Imig) and *t*_½_∼120 (Vela) (Fig. 3). The splenic CRIM-SA of Vela or Imig in 5-wk 9 V/null mice was lower (0.2–0.8×10^6^ nmoles/h/mg enzyme protein) than that in 20-wk 9 Vnull mice; at 5 wks there were no significant differences in CRIM-SA between Vela and Imig (*p* = 0.114).

Overall, as summarized in Table 4, the majority (60–70%) of injected GCase was localized to the liver in either age group. Spleens had an uptake of ∼2–3% of injected enzymes, but the peak activities were ≥2-fold of WT levels at 20 min post-injection. In comparison, less than 0.2% of injected activities were recovered in lung. These results indicated that the enzymes were delivered mostly to the liver and that a substantial portion of enzyme (30–40%) was distributed to other organs or was inactivated/denatured.

### Therapeutic effects

Therapeutic effects were evaluated in 9 V/null mice that were treated weekly with 5, 15, or 60 U/kg/wk of Vela and Imig for either 4 or 8 wks. Mice were sacrificed one week after the last injection, and sera and organs were collected for serologic studies, GCase activities, GC levels, and quantification of storage cells. At one week after the last injection, the GCase activities in liver, spleen, and lung had returned to background levels (not shown).

#### GC levels in liver, spleen, and lung

**Figure 4:**
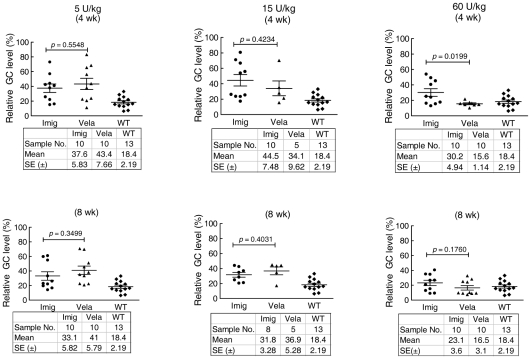
Effect of Vela or Imig on GC levels in liver. 9 V/null mice were injected with 5, 15, or 60 U/kg/wk of Vela (▴) or Imig (•) for 4 wks (top panels) or 8 wks (bottom panels). GC liver levels were quantified by LC/MS and expressed as the percentage of the saline-injected group. WT mice (♦) are provided for reference. GC values in accompanying data tables represent the mean ± SE from 5–13 mice in each cohort. Liver GC values for Vela- or Imig-treated mouse cohorts were analyzed by *t*-test.

GC levels in liver by week 4 or 8 decreased by 50–70% in mice receiving 5 or 15 U/kg/wk of Vela or Imig weekly (Fig. 4) compared to saline-injected controls. In comparison, GC level decreases with 60 U/kg/wk were ∼70% with Imig and ∼85% with Vela at week 4, and by ∼80–85% for both enzymes at 8 wks (Fig. 4). With 5 or 15 U/kg/wk doses, no significant differences were noted between the Vela and Imig groups (*p*>0.05). However, at 15 U/kg/wk doses, the Vela-injected mice showed a trend toward greater (∼66%) reductions of GC levels compared to ∼55% in Imig injected mice (p = 0.42). By 8 wks, this difference disappeared as WT GC levels were achieved with either GCase. With 60 U/kg/wk, incrementally greater decreases in GC levels were observed at 4 wks, and the reduction to near-WT levels was again achieved using Vela, whereas this was not the case with Imig (*p* = 0.0199). WT GC levels were achieved and maintained with either GCase by 8 wks at this dose. There was a ∼20% greater GC reduction in the 4- and 8-wk groups receiving 60 U/kg/wk compared to that in the 5 and 15 U/kg/wk groups; the results represent a dose response trend (*p*>0.05). The decrease to WT levels by 8 wks obscures any additional differences that might exist between Vela and Imig.

**Figure 5:**
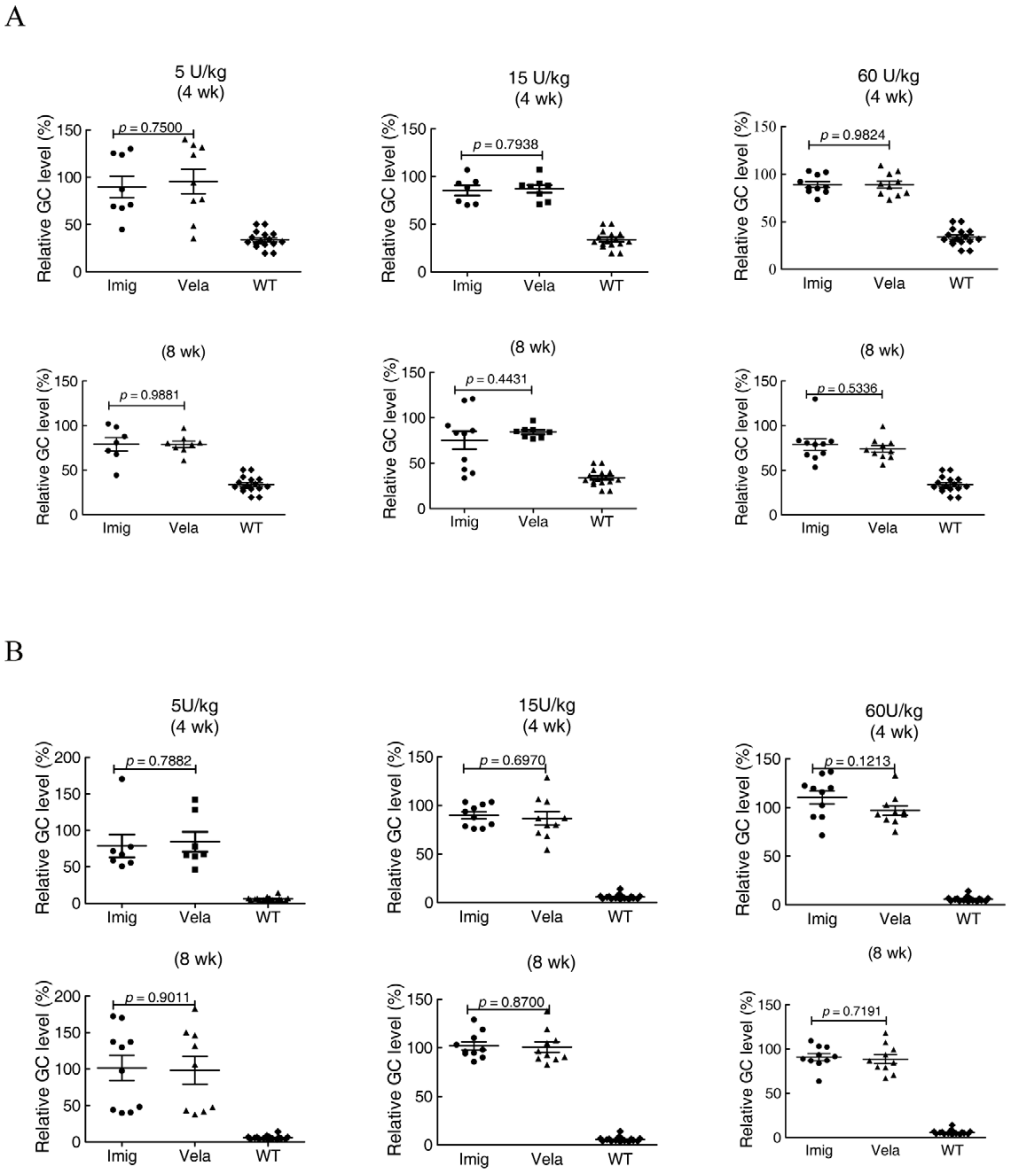
Effect of Vela or Imig on GC levels in Spleen (A) and Lung (B). The graphs contain the GC values (mean ± SE) from 7–16 mice in each dosing cohort relative to the values from saline injected mice. The symbols and analysis methods are the same as in Figure 4.

For all three dosing groups, the GC levels in spleen were reduced by ∼10–15% at 4 wks and by ∼20–30% at 8 wks (Fig. 5A). WT levels were not achieved with any dose, at either time or with either enzyme. In each dosage group, the reductions of GC levels by Vela and Imig were similar (all *p*>0.05). In lung, the GC lipid levels were unchanged at the doses of 5 and 15 U/kg/wk (Fig. 5B) with either preparation. However, a suggested trend toward ∼10% decrease of GC was observed in lung at 60 U/kg/wk for either enzyme preparation at 8 wks, but the results were variable within and among groups (Fig. 5B).

#### Storage cells

**Figure 6:**
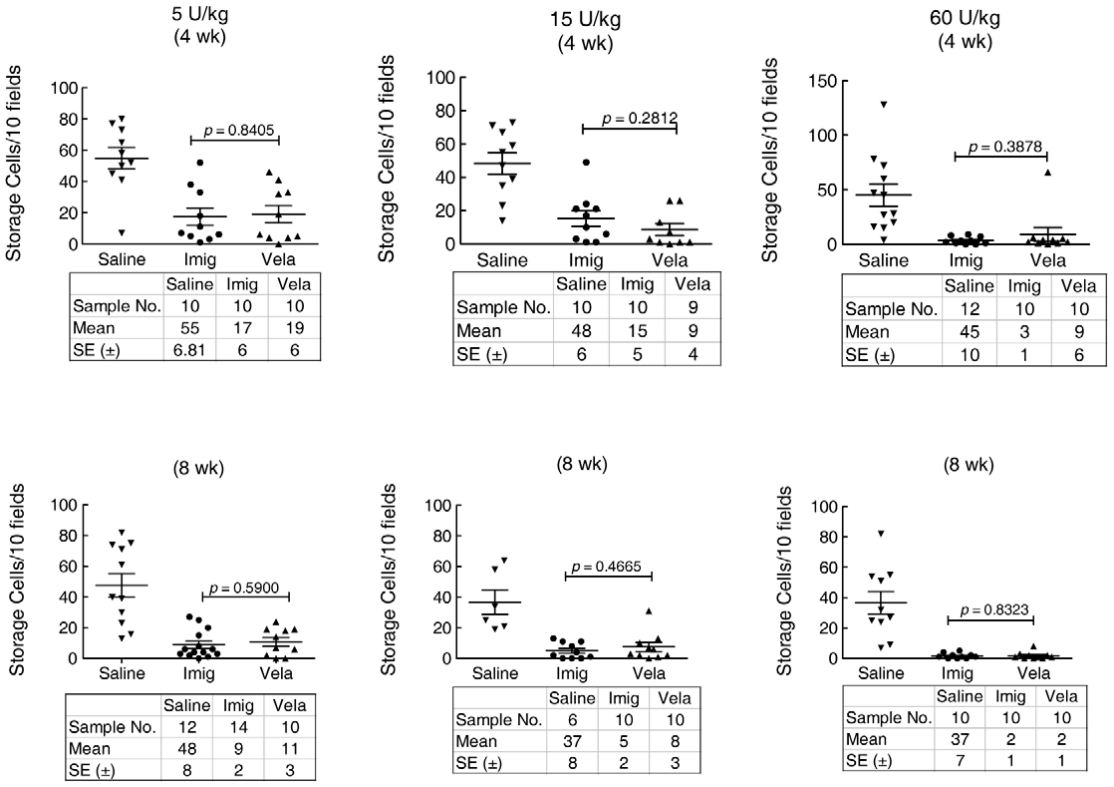
Reduction of liver storage cells with Vela or Imig administration. Liver storage cell quantification was done after administration of various doses of Vela or Imig for 4 or 8 wks. Liver H&E sections from these mice were examined by light microscopy, and storage cells in 10 independent fields from each section were counted. The mean (±SE) values are shown in the data tables. Liver sections from saline-treated mice (▾) were processed in parallel. Storage cells in Vela (▴) or Imig (•) treated mice (n = 6–14 mice) in each cohort were analyzed by *t*-test.

Lipid-laden macrophages—Gaucher cells—are a hallmark of Gaucher disease. H&E-stained liver sections showed that storage cells were large, engorged, and pale in color (not shown). In quantitative comparison to the saline control group, the number of storage cells in either the Vela- or Imig-treated mice was significantly decreased in all dosage groups, and the degree of change correlated with the dose increment and length of therapy (Fig. 6). Although significant changes from controls were evident at all doses, times, and enzyme preparations, 60 U/kg/wk nearly eliminated these cells in liver by either 4 or 8 wks (Fig. 6). No significant differences were evident in liver storage cell numbers between Vela and Imig at any dose (*p*>0.05).

To evaluate the storage cells in the lung, the sections were processed for immunohistochemistry using rat anti-mouse CD68 antibody, a macrophage membrane antigen [38]. The lipid-laden storage cells in lung were generally large cells that stained brown (CD68-positive), indicating the macrophage origin of these lipid-laden storage cells. The numbers of storage cells in Vela- and Imig-treated 9 V/null lungs at any dose were not different from saline control (data not shown).

The GC levels and numbers of storage cell showed concordant changes with enzyme administration. However, GC levels in the liver were still above WT levels at 8 wks of 60 U/kg/wk, even though the liver was essentially cleared of storage cells, indicating some excess GC was present in cells that are not typical Gaucher cells. Such quantitative correlations could not be made in the spleen as representative matched samples were not available, since GC and enzyme determinations consumed nearly the entire spleen. The numbers of storage cells in a few available sections appeared decreased, but not absent. The spleen GC levels remained significantly elevated even after 8 wks of 60 U/kg/wk.

### Antibody responses

**Figure 7:**
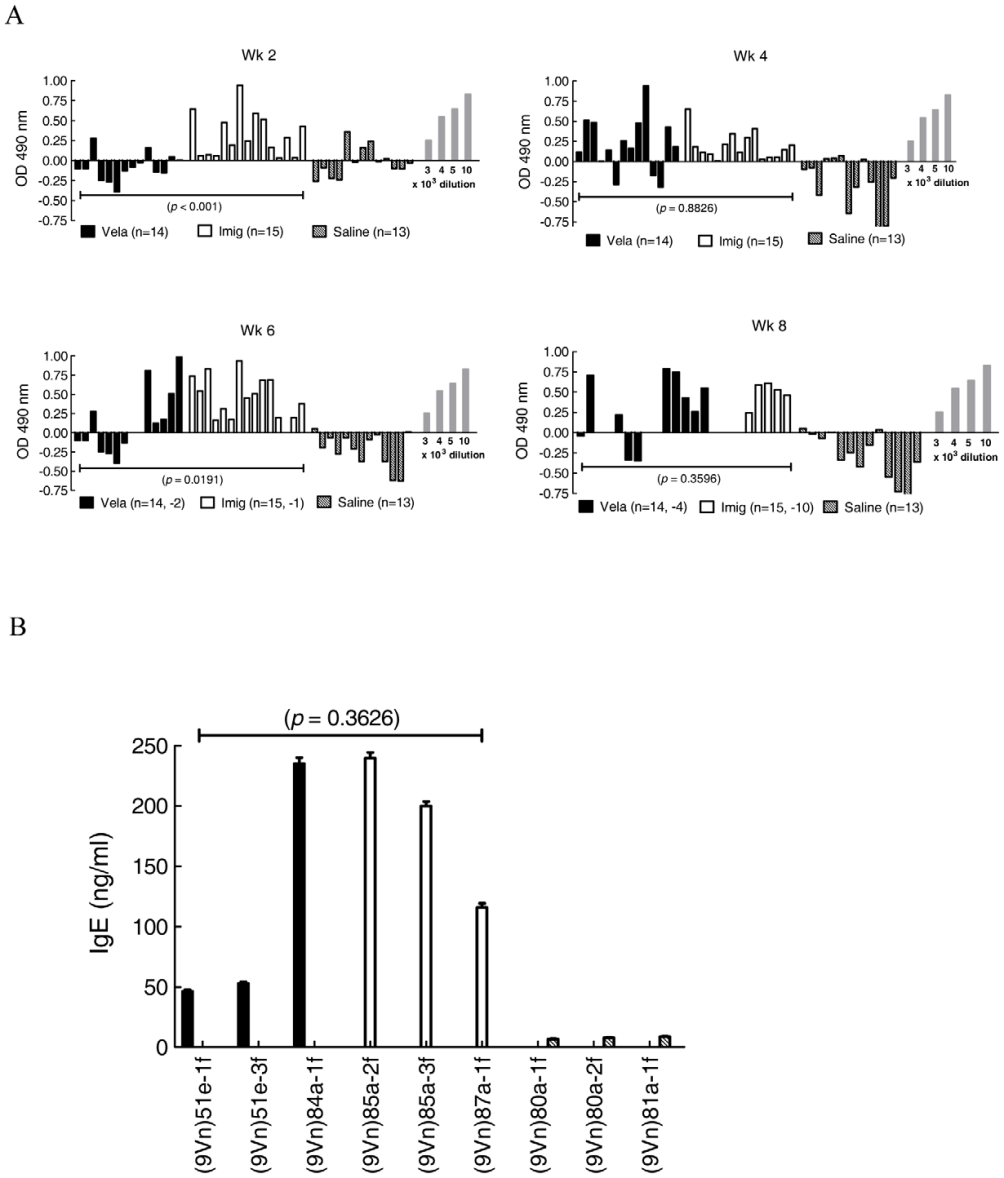
Anti-human GCase IgG (A) and IgE (B) in Vela- or Imig-treated 9 V/null mice. (**A**) 9 V/null mice that received Vela (solid bars) or Imig (open bars) were bled prior to every second injection, and mouse anti-human GCase IgG levels were assessed by ELISA. The OD 490 nm (Y-axis) readings from each mouse (X-axis) are presented. The number of tested (n) in each group is listed below the graph with the number of mice that died indicated as (-n), e.g., (n = 15, −1) means that 15 mice were assayed and that one mouse in that group had died from the injection related reaction, and the empty lane on the graph represents the death of those mice from which no serum was obtained. Assays were in triplicate; control samples were from saline-injected (hatched bars) mice and were conducted in parallel. The standards (grey bars) were determined with mouse anti-human GCase antiserum (3–10×10^3^ dilutions) and were used as a reference. (**B**) Total serum IgE levels in Vela- or Imig-treated mice that experienced acute immunological/anaphylactic reactions. Shown are results from serum samples from three mice that died with apparent anaphylactic reactions following 4 or 5 injections of Vela (solid bars) or Imig (open bars) with a 60 U/kg/wk dose. The values were quantified using pure mouse IgE protein as a standard and plotted as ng IgE/ml. Assays were conducted in triplicate; control samples were from saline-injected mice (hatched bars) and were conducted in parallel.

To evaluate the immune responses during ERT, serum for anti-human GCase IgG was obtained every 2 wks during the 8-wk trial. Using our mouse anti-human GCase antiserum, the titers of developing anti-human GCase were determined in each mouse in a standardized manner. Importantly, because of some differences in reactivity, Vela or Imig were used as antigen controls for the respective samples from mice. For the 5 or 15 U/kg/wk doses, serum anti-GCase IgG signals toward either Vela- or Imig-injected mice were at background or very low levels from each bleed (not shown). In the mice receiving the 60 U/kg/wk dose, serum anti-GCase IgG levels increased ∼3-fold (at ∼3×10^3^ dilution) above background by the 4th injection in >50% of Vela- and Imig-injected mice (Fig. 7A).

With additional injections, IgG positivity increased in all dosing groups and several mice died. With the 5 or 15 U/kg/wk doses only a few mice died, whereas with 60 U/kg/wk doses, 9/42 Vela-injected and 20/59 Imig-injected mice died following injections during the 4- to 8-wk period (Table 5). Prior to death, these mice became immobile and their lips/tails became cyanotic. Most mice died within 30 to 60 min post-injection. However, placing treated mice in an oxygen chamber for 20 to 30 min after injection led to the survival of most of the animals. The mice that succumbed were immediately dissected, and sera were analyzed for IgE titers and histology. The H&E sections from lung, heart, spleen, kidney, and brain did not show evidence of an acute inflammatory response. Total serum IgE levels from these mice injected with Vela or Imig were 46.4 to 234.9 ng/ml (n = 3) or 115.8 to 239.6 ng/ml (n = 3), respectively (*p* = 0.3626). In comparison, saline-injected 9 V/null serum values were 6.8 to 8.8 ng/ml (n = 6) (Fig. 7B).

**Table 5:** Injection-related deaths^A^.

Enzyme	No-oxygen		Oxygen^B^		Total number	
	Mice^C^	Death (%)^D^	Mice^C^	Death (%)^D^	Mice^C^	Death (%)^D^
Vela	31	7 (22.6%)	11	2 (18.2%)	42	9 (21%)
Imig	31	15 (48.4%)	28	5 (17.9%)	59	20 (34%)

A 9 V/null mice were injected with 60 U/kg of Vela or Imig, once a week for 8 weeks; the mouse death number and percentage (%) of total injected mice in 8-wk courses are presented.

B Oxygen chamber was applied for minimum 20 min to the mice receiving Vela or Imig.

C The number of injected mice in each cohort;

D The number and percentage of dead mice from total injected mice.

## Discussion

The advent of enzyme therapy for Gaucher disease about two decades ago led to a major change in the care and health outcomes of affected individuals. The use of regular infusions of mannosyl-terminated GCase has led to remarkable improvements in the overall health, and visceromegaly, organ dysfunction, and bony complications experienced by people affected with Gaucher disease type 1. However, the use of this expensive therapy has led to controversies surrounding the cost-benefit and the degree of reversibility of specific manifestations. Such controversies continue to be based on clinical measurements of organ volume, MRI changes, X-ray alterations, and changes in blood counts or presumptive biomarkers such as chitotriosidase. Pharmacokinetic or pharmacodynamic studies have been lacking in humans or animal models to evaluate the effects on disease processes. Comparative dose-finding schedules have not been evaluated in humans because of a reluctance to sample appropriate tissues, and due to the ethical issues associated with obtaining such tissues for determination of differential biochemical and histological effects. Here, mouse analogues of human Gaucher disease with tissue involvement, including the presence of Gaucher cells and excess GC storage, were used to compare two preparations of mannosyl-terminated GCases for treatment effects and dose response as well as pharmacokinetics and pharmacodynamics. Although not strictly comparable to humans, the differential immunological responses of the mice to the infusions of human enzymes were compared with the two agents.

The two preparations of mannosyl-terminated GCase are highly similar for *in vitro* kinetic properties and interactions of their active sites with substrates and inhibitors. The amino acid sequences of the two enzymes differ by one amino acid at position 495, in which Imig contains a histidine (R495H) substitution for an arginine (R495), the naturally occurring amino acid that is present in Vela [39]. The present results are consistent with those from previous studies in which GCase was expressed in the baculovirus system [28], [29]; the R495H had no influence on the *in vitro* kinetic properties or stability of the enzyme relative to the R495 GCase [28], [29]. In particular, the kinetic constants for interaction with either reversible (iminosugars) or irreversible inhibitors (CBE and 2,4-DNPFG) were similar. Of particular interest was that the GCase's interactions with the very potent reversible inhibitor IFG (Ki∼20 nM) were similar. This inhibitor is known to induce a significant conformational change in loop 1 of GCase, indicating that at least at this level, the two enzymes have similar movements [35]. *In vitro*, enhancers of GCase activity, i.e., saposin C and the negatively charged phospholipids showed no differences between these GCases or those previously reported [29], [40]. The catalytic rate constant of Vela was consistently greater (6–10%) than that for Imig based in the quantification of CRIM or active sites using the single-turnover substrate DNPFG. These differences were not significant (*p* = 0.85) and there was some batch-to-batch variation due to storage.

A significant difference in structure between the Imig and Vela is the presence of a greater number of mannosyl residues attached to the latter as a result of the kifunensine treatment of the producing cells. This molecule inhibits ER α-mannosidase I and prohibits the cleavage of extended mannosyl structures present on the nascent GCase prior to its modification and remodeling in the Golgi [41], [42]. The predominant number of mannoses on Vela is 9 [43]. In comparison, Imig contains a smaller number (∼3) of mannoses that are exposed by sequential exoglycosidase treatment following expression in CHO cells and purification [44]. These differences in oligosaccharides had no effect on the *in vitro* stabilities or the kinetic properties of either enzyme. These results are similar to those obtained previously [44].

The pharmacokinetic and pharmacodynamic studies show essential similarities between Vela and Imig administered as intravenous boluses. The majority of injected enzyme can be recovered from the liver (≥60%) and smaller amounts in the spleen. Immunofluorescence showed that the cellular distribution/concentration in the liver was to/in interstitial cells, including endothelial cells and Kupffer cells, with low-level discrete fluorescence signals over the hepatocytes. The distribution of mannosyl-terminated GCases within livers of normal mice showed parenchymal hepatocyte as well as endothelial cells and Kupffer cell uptake [9], [44], [45]; this is likely the case here also. Although not formally evaluated, the concentration of human GCase fluorescence signals in the periportal regions and interstitial areas indicates a preferential, but certainly not exclusive, uptake into endothelial and Kupffer cells. This is reflected in the nearly complete disappearance of storage cells in liver, but GC levels remaining elevated at 5 and 15 U/kg/wk with either enzyme even at 8 wks of treatment. Previous studies with acid lysosomal lipase that was mannosyl-terminated showed that even in the absence of the macrophage mannose receptor, mannosyl-terminated enzymes can be delivered to Kupffer cells, endothelial cells, and hepatocytes in affected mice [45]. No differences were observed in tissue distribution between Vela and Imig using immunblot assays, nor did there appear to be significant preferential differences by immunofluorescence in cellular uptake. Of significance is the essential lack of intravenously administered GCase recovery from the lungs of the affected mice. In these 9 V/null mice, large numbers of storage cells accumulate interstitially and in the alveoli. Very little, if any, of the intravenously administered enzymes could be detected in the lungs of these affected mice at any time point. This is consistent with the anecdotal observations of persistent alveolar storage cells in lungs of a Gaucher disease patient even after 15 months of ERT via right atrial catheters [24].

Following single intravenous administrations of equivalent amounts of enzyme to these affected mice, the half-lives of disappearance for the enzymatic activity and the GCase protein were determined in a variety of tissues. Both showed similar disappearances of enzymatic activity and protein in all the tissues. In plasma where GCase is known to be unstable due to the exposure to pH 7.4, the catalytic rate constant (approximated by CRIM-SA) of the enzymes decreased with time. By ∼20 min after injection, the intrinsic activity of the enzyme remaining in plasma was <5% of that in the original injected doses for either enzyme. It should be noted that with time, the amount of enzyme protein and activity in plasma becomes very small making accurate assessments difficult at later time points. The disappearances of GCase activity and protein in the tissues were similar between the two enzyme preparations. Of importance, even though the half-lives in liver and spleen were 1–1.5 h, the exogenous GCases in liver and spleen remained stable in terms of their intrinsic catalytic activity as assessed by CRIM-SA. This implies that once delivered to the lysosomes of the tissues, the administered GCases remain in a stable catalytic form with the same intrinsic activity as the administered enzyme for significant periods of time. Thus, although the amount of enzyme decreases significantly in the tissues, the enzyme that retains normal activity can contribute significantly to the amount of residual activity in these mice.

Also, only minor differences were determined between mice at 5 wks compared to those with more advanced disease at 20 wks of age. These results imply that the degree of disease involvement, particularly of the liver, has small effects on the distribution or degradation of the administered enzyme. Of note was the more rapid disappearance of either administered GCase activities and proteins in the 5- vs. the 20-wk mice. A small difference was also appreciated between the two GCases, with Vela being cleared somewhat more rapidly from the 5-wk livers than Imig. No difference in this parameter was noted between these GCases in the 20-wk mice. For either enzyme, the activity and protein returned to essentially baseline levels by 20–42 h. Immunologically, the human enzyme could be distinguished from the intrinsic mouse enzyme by the use of a specific antibody that does not react with the small amount of mouse enzyme present in this model. These results suggest that, although minor, the more rapid clearance of the GCases from the liver in the less involved mice could impact the continued improvement in patients, e.g., as their overall disease, particularly the liver involvement, is reversed, the length of time that their organs experience a therapeutic level of the GCases could be shortened and the rate of reversal could slow [8], [18].

Significant discussions have been devoted to the dose response of patients to the GCase therapy. Recent studies based on registry data and direct clinical observations have resolved the issue of dose response to Imig by showing incremental improvements in clinical parameters based on increasing doses of Imig [18], [46]. However, essentially no data are available on the effects at a tissue level of biochemical or histological aberrations for different doses. The accumulation of Gaucher cells and GC in 9 V/null mice provided the opportunity to evaluate dose response at a tissue level and also to conduct comparative studies of the two GCases at three different doses; the dose range was 12-fold (5–60 U/kg/wk). At a histological level, when administered to either 5-wk or 20-wk mice, a difference in the quantitative liver (*p* = 0.28–0.84) or lung (data not shown) cytopathies was not observed when comparing the effects of either GCase. Over a period of 8 wks with weekly dosing, essentially a complete clearance of GC and Gaucher cells from the liver and significant clearance of GC from the spleen were observed. This effect was dose- and time-incremental, so that by 4 wks a significant change had occurred; This was most evident in the liver at 60 U/kg/wk where Vela reduced GC to WT levels, whereas Imig did not (*p* = 0.0199). By 8 wks of treatment this difference was no long apparent (*p* = 0.176) as Imig had, by then, reduced the GC in the liver to WT levels. The lungs, particularly the alveolar Gaucher cells, were not cleared to any significant extent by either enzyme. A difference of ∼10% in cellular clearance was observed with Vela compared to Imig. However, there was substantial variation in cell count number, and this difference in Gaucher cell clearance was not significant. A lack of effect in the lungs is not unexpected, since human studies showed persistence and progression of alveolar Gaucher cells even with Imig infusions directly into the right atrium, thereby providing direct pulmonary circulation infusions of very high amounts of enzyme [24]. The basis for this lack of alveolar effect is unclear, but may be due either to enzyme not entering the alveolar space or to the enzyme being carried into the alveoli by cells with long residence times, during which the administered GCase may be degraded. The absence of enzyme would leave the alveolar macrophages deficient in GCase for the majority of their life spans. The source of the lung substrate is unknown, but is probably due to local synthesis within the bronchiorespiratory tree.

Concordant with the changes in Gaucher cell number within the tissues were decreases in GC content. In both liver and spleen, significant decreases in GC content were observed at any of the doses. As with the cell counts the change in lipids was proportional to the dose given with the higher doses showing more substantial clearance than the dose at 5 U/kg/wk. The 5 U/kg/wk effect was not statistically different between the two enzyme preparations. However, with 15 and 60 U/kg/wk at 4 wks, Vela affected greater degrees of decrease of GC in the liver; the latter dose was statistically significant at 4 wks. This effect at 4 wks is the only statistically significant difference at a tissue or a biochemical level between these two enzyme preparations. Importantly, the effects in liver and spleen were significant at all three dosage levels, and the lack of effect in the lungs was the same for all three dosages. These studies cannot determine if longer-term therapies or different modes (e.g., slow infusion) of administration would permit the clearance of Gaucher cells and GC from the lung, but the human studies suggest that even long-term intravenous administration on an intermittent basis does not lead to clearance in the lungs. Evidently, alternatives for this rare manifestation in humans will have to be developed. To this end, the clearance of the alveolar Gaucher cells in the 9 V/null mouse receiving a potent GC synthase inhibitor Genz 112638, i.e., substrate synthesis inhibition therapy, has been observed [47].

Not unexpectedly, the mice receiving human GCase developed antibodies to this antigen in almost all circumstances. IgG was detected in many mice, and a significant number of mice developed an anaphylactic-like reaction after several doses of either enzyme. Indeed, IgE was present in a number of mice from whom sera were available, suggesting an anaphylactic mechanism. The clinical parameters of cyanosis—difficulty breathing and tachycardia—were consistent with this. Although there were no statistically significant differences in antibody (IgG or IgE) positivity rates (*p* = 0.3636), there was a significant difference in the number of mice that died during the studies while receiving Imig compared to Vela. Whether this increased death rate was due to the enzyme administration procedure or some immunologic reaction to Imig as compared to Vela is not known. However, these studies suggest that at least in the mouse, there may be some antigenic recognition differences between the two enzyme preparations leading to elicitation of the mouse antibody response and culminating in the death of some of the mice. Not unexpectedly, the antibody positivity rate in these mice was much greater than that seen in humans, which has remained at ∼13–15% for Imig for the past ∼20 years [32]. If transferable to humans with Gaucher disease type 1, Vela might be expected to have lower antibody positivity rates. Thus, while these antibody responses are not exactly comparable between humans and mice, they are suggestive of potential differences that might be elicited by either the enzyme or the other components in the clinical preparations of the supplied enzyme.

In summary, these studies show similar effect of Vela and Imig at several different doses, at a therapeutic level, and at different ages with differing degrees of involvement. There do not appear to be any particular adverse effects of the administered enzyme secondary to antibody production, although there were IgE-mediated reactions in these mice receiving human GCases leading to death. Furthermore, these studies demonstrate clearly that both enzymes achieved similar therapeutic effects, with the doses and route of administration used here, and at different ages or degrees of involvement of the mice. Significantly, the lungs are not altered in their degrees of involvement by enzyme therapy at any dose over an 8-wk period in this mouse model.

## Materials and Methods

### Materials

The following were from commercial sources: Imiglucerase (Imig, Lot#A7021H04, Genzyme, Cambridge, MA). Velaglucerase alfa (Vela, Lot#FEC06-002, HGT/Shire, Cambridge, MA). Preservative-free, sterile water (Hospira, Lake Forest, IL). 0.9% sodium chloride for injection (Abraxis Pharmaceutical, Schumburg, IL). Conduritol B epoxide (CBE), deoxynojirimycin (dNM) and sodium taurocholate (TC) (Calbiochem, San Diego, La Jolla, CA). 4-methylumbelliferyl-β-D-glucopyranoside (4-MU-Glc; Biosynth AG, Switzerland). Isofagomine-HCl (IFG), C_4_-, C_9_-, and C_12_-dNM (Toronto Research Chemicals, Toronto, Canada). 2,4-dinitrophenyl-2-fluoro-2-deoxy-glucopyranoside (DNPFG), Triton X-100 (TX), castanospermine (CS), and SIGMAFAST OPD (Sigma, St. Louis, MO). Brain phosphatidylserine (BPS), C_12_-NBD, and C_12_-NBD-glucosylceramide (NBD-GC) (Avanti Polar Lipids, Alabaster, AL). Cathepsin D (EMD Biosciences, Gibbstown, NJ). Isoflurane (Abbott Lab, Abbott Park, IL). Heparinized microhematocrit capillary tubes (Cardinal Health, Dublin, OH).

Hybond ECL Nitrocellulose Membrane (Amersham, Piscataway, NJ). 4–12% gradient Bis-Tris Gel (Invitrogen, Carlsbad, CA). BCA Protein Assay Kit, Peroxidase-Conjugated Goat Anti-rabbit IgG, M-Per Mammalian Protein Extraction Reagent, ECL Kit, and Purified Mouse IgE (PIERCE, Rockford, IL). Mouse IgE ELISA Kit (BD, Franklin Lakes, NJ). HRP-conjugated goat anti-mouse IgG (Dako, Denmark). Rat anti-mouse CD68 and goat anti-rat-HRP (Serotec, Raleigh, NC). Goat anti-rabbit biotinylated and streptavidin-Alexa Fluor-610 (Molecular Probes, Irvine, CA).

### Methods

#### *In vitro* enzymology studies

Vela and Imig were compared for kinetic properties as described [29]. Briefly, the standard GCase activity assay contained final concentrations of 50 mM citrate/phosphate, pH 5.5, 0.25% TC, 0.25% TX, and 4 mM 4MU-Glc in 200 µl. The reactions were stopped after incubation (30–60 min, 37°C) using 0.9 M ethylenediamine, pH 11.0 (100 µl). Standard curves for 4-MU fluorescence yield were generated for each assay with Ex/Em 460/515 nm (Molecular Dynamics SpectraMax GeminXS spectrofluorometer). V_max_ and K_m_ were calculated from Lineweaver-Burke equations using several concentrations (0.25–10 mM) of 4MU-Glc. For the reversible competitive inhibitors, K_i_ was calculated from IC_50_ = K_i_ (1+ [I]/[K_m_]). The IC_50_ or K_i_ values for various inhibitors (dNM and its derivatives, CBE, CS, IFG, DNPFG) were determined using 4MU-Glc as substrate. Activities with the NBD-GC substrate were as described [48]. Enzyme assays with saposin C (0–500 nM/0.5 µM BPS) and BPS (0–2 µM) were as described [29], [40]. Saposin C was expressed in *E. coli* and purified as described [40]. For thermostability assessments, equal amounts of Vela and Imig activities were added to 50 mM citrate/phosphate, pH 7.4, with or without human/mouse serum, and incubated for 0–240 min at 25 or 37°C. The pH optimum profiles were developed using 50 mM citrate/phosphate, pH 4.4–7.2. Protease susceptibilities of the two GCases were evaluated by cathepsin D (1–2 µM) digestion in 25 mM sodium acetate, pH 4.8, 50 mM NaCl, 1.25 mM EDTA, 1.25 mM DTT using 200 ng of Vela or Imig at 37°C for 4 h [29]. All assays were conducted in triplicate and repeated at least twice. The amounts of intact enzyme were determined from density measurements on immunoblots and were compared to the initial amounts. The ratios of intact enzyme with and without cathepsin D were used as indicators of the enzyme's proteolytic susceptibility.

#### Mice with point-mutated GCase

D409V/null (9 V/null) mice and WT littermates were of mixed genetic backgrounds [33]. The CCHMC Institutional Animal Care and Use Committee (CHCMC IACUC) reviewed and approved these studies under protocol 7C02017. All mice were housed under pathogen-free conditions in the barrier animal facility and according to IACUC standard procedures at Cincinnati Children's Hospital Research Foundation. Mice were monitored daily and weighed weekly. In visceral tissues, the GCase activities were ∼1–5% of wild-type levels. Mice at 5 and 20 wks of age were used for these studies.

#### Enzyme preparation for injection

Vela and Imig were reconstituted with sterile water under sterile conditions according to manufacturers' instructions. The reconstituted concentrations of Vela and Imig were 100 U/ml and 40 U/ml, respectively. A unit (U) of GCase activity is 1 µmole/min of 4-MU cleaved from 4-MU-Glc at 37°C, in 50 mM citrate/phosphate, pH 5.5, in the presence of 0.25% TC and 0.25% TX [28]. Aliquots of reconstituted enzymes were stored at −80°C that were diluted with 0.9% NaCl just prior to injection. Vela and Imig activities were determined on the day of injection and were used for pre- and post-injection activity calculations.

Enzymes were injected intravenously via tail vein under sterile conditions using a U-100 insulin syringe with a 28Ga needle (Becton Dickinson). Mice were anesthetized with isoflurane. For the PK/PD studies, two age groups (5 and 20 weeks old) of 9 V/null mice (3 mice per cohort) were injected with 60 U/kg of Vela or Imig. Samples were obtained at 2, 5, 10, 20, and 40 min and at 1, 2, 4, 18, and 42 h. For therapeutic studies, 9 V/null mice were administered doses of 5, 15, or 60 U/kg/wk of Vela or Imig weekly for 4 or 8 wks.

#### Tissue and serum collection and GCase assay

Blood samples from anesthetized mice were taken from the retro-orbital plexus. Serum was separated from clotted blood (0.5 h, 4°C) by centrifugation at 12,000 rpm (15 min). Mice were then euthanized by CO_2_ narcosis and perfused with normal saline (2 to 3 times blood volume) prior to tissue harvest. Tissues (liver, spleen, lung, and brain) were harvested immediately, rinsed with saline, blotted dry, snap frozen, and stored at −80°C for enzyme, lipid, histological, and immunoblot analyses.

Tissue lysates were prepared as described [23]. Protein concentrations were determined using the BCA Protein Assay Kit. For GCase activity assays, tissue lysates were pre-incubated in the presence or absence of CBE (2 mM, 30 min, room temperature) prior to the addition of 4MU-Glc substrate to estimate the amount of tissue non-acid β-glucosidases that cleave 4-MU-Glc [28], [29]. For serum GCase activity assays, 5 µl of freshly collected serum was diluted in 10 ml of saline, and 5–25 µl aliquots were used for assay. GCase activities are expressed as nmoles/h/mg of tissue protein or nmoles/h/ml of serum. Control non-injected or saline-injected and WT mouse tissues were processed in parallel.

#### Immunoblots, CRIM, and CRIM-SA

CRIM-SA was based on the amount (ng) of intact GCase (M_r_∼60,000 to 63,000) CRIM quantified from immunoblots using polyclonal antibody [41]. For these analyses, tissues were extracted (1∶5, wt/vol) in M-Per with a dounce homogenizer (10–12 strokes). Equal amounts of supernatant protein from tissues or equal volumes of serum at each time point were subjected to SDS-PAGE. For immunoblots, membranes were pre-treated with 3% BSA in PBS-0.05% Tween-20 (1 h) incubated with the rabbit anti-human GCase antibody and, then, peroxidase-conjugated goat anti-rabbit IgG in PBS-0.05% Tween-20. The known amounts of Vela or Imig standard (0.5–6 ng) were mixed with serum aliquots or tissue lysates from saline-injected 9 V/null mice and loaded onto each gel for quantification standards and as background controls. The immunoblots were developed using ECL kits, and the amount of human GCase cross reacting immunological material (CRIM) in each lane was quantified using ImageQuant5.2 software. Known amounts of Vela or Imig proteins were used as standards for the respective samples from injected mice.

#### Lipid analyses

GC levels in liver, lung, and spleen from 9 V/null and WT mice were quantified by LC/MS-MS (Lipidomic Core, University of South Carolina). The GC levels were normalized to protein in the same lysates used for GCase activity and expressed as the percentage change relative to the GC levels of matched samples from saline-injected mice. WT lysates were used as a reference. The Student's *t*-test was used for statistical comparisons.

#### Storage cells

Duplicate tissue sections were fixed in 10% formalin for hematoxylin and eosin (H&E) staining and in 4% paraformaldehyde-PBS, pH 7.4 for immunohistochemistry with CD68 antibody [49] and examined using a Zeiss microscope equipped with SPOT Advance software (SPOT Diagnostic Instruments, Inc.). Large pale cells (≥5 µm, WT<5 µm) in H&E-stained liver sections and CD68-positive macrophages in lung sections were counted manually in 10 randomly selected fields (305 µm ×228 µm/field) from each mouse. Mice (n = 6–14) in the Vela-, Imig-, or saline-treated groups were used, and the resultant data were analyzed using the Student's *t*-test.

#### Detection of mouse anti-Vela or -Imig IgG and total IgE

For the 8-week therapeutic study, 9 V/null mice were dosed with 5, 15, or 60 U/kg/wk of Vela or Imig and were bled every other week, before enzyme injections, for GCase antibody analyses. ELISA was used to determine the mouse anti-GCase IgG levels. Immulon-4 plates (96-well) were coated with 100 ng of Vela or Imig and processed [50]. Mouse serum samples (100 µl, 1∶4 dilutions) were used for analyses with pre-immune serum as negative controls. Standard curves were generated with mouse polyclonal anti-human GCase antiserum (1∶50 to >1∶100,000 dilution) on the same plates. The reaction was stopped by addition of 3 M H_2_SO_4_ (50 µl) per well and read at 490 nm (Molecular Dynamics SpectraMax GeminXS). The absorbance values were expressed as endpoint titers of each sample and normalized using the standard curve. For detection of mouse total serum IgE, the mouse samples were analyzed with the Mouse IgE ELISA kit as above [50]. Biotinylated anti-mouse IgE monoclonal antibody and streptavidin-horseradish peroxidase conjugate (SAv-HRP) were used for detection. Purified mouse IgE was used as standard for quantification.
